# The contemporary trend in worsening prognosis of pancreatic acinar cell carcinoma: A population-based study

**DOI:** 10.1371/journal.pone.0243164

**Published:** 2020-12-17

**Authors:** Nie Duorui, Bin Shi, Tao Zhang, Chuyao Chen, Chongkai Fang, Zhijun Yue, Peng Wu, Zhiming Wu, Xuewu Huang, Meng Li

**Affiliations:** 1 First Clinical Medical College, Guangzhou University of Chinese Medicine, Guangzhou, Guangdong, China; 2 Cancer center, Guangzhou University of Traditional Chinese Medicine First Affiliated Hospital, Guangzhou, Guangdong, China; 3 Department of Oncology, Shenzhen Hospital (Futian) of Guangzhou University of Chinese Medicine, Shenzhen, Guangdong, China; Texas Tech University Health Science, Lubbock, UNITED STATES

## Abstract

**Background:**

Primary acinar cell carcinoma (ACC) is a rare exocrine tumor of the pancreas with unclear clinical characteristics. Our goal was to determine the incidence and update the clinical characteristics and outcomes of ACC.

**Methods:**

Through the Surveillance, Epidemiology, and End Results (SEER) database, we identified 252 patients with the latest diagnosis of ACC (2004–2016). The age-adjusted incidence (AAI) was calculated using the SEER*Stat Software version 8.3.6. The Kaplan–Meier method was used to draw survival curves and differences among them were compared by the log-rank test. Cox proportional hazards models were used to evaluate factors that had independent predictive effects on the overall survival.

**Results:**

The AAI of pancreatic ACC was on the rise with the mean age at diagnosis of 63.79±14.79 years. Most patients (15.9%) had poorer differentiated tumors. The patients presented with distant stage were 54.4% compared with 53.1% between 1988 and 2003. The 1-, 2-, and 5-years survival rates for pancreatic ACC patients were 53.5%, 34.6%,17.5%, respectively (compared with 78.5%, 67.0%, and 42.8%, between 1988 and 2003). The multivariate COX analysis showed that the patient's age, surgery, chemotherapy, and summary stage, but not marital status were independent prognosis factors for ACC.

**Conclusions:**

Pancreatic ACC is a highly malignant tumor with an increasing incidence in recent years. The rate of distant metastasis is increasing and the survival rate is worse than in the past, suggesting that it may require more aggressive treatment and follow-up. Surgery, radiotherapy, and chemotherapy are all effective treatments, but prospective studies are still needed to verify them.

## 1. Introduction

Acinar cell carcinoma (ACC) is a rare exocrine tumor that can occur anywhere in the pancreas, accounting for 1%-2% of all pancreatic tumors [1, 2]. ACC occurs most frequently in white males and is usually bimodal distribution in age with peaks in childhood and adulthood [3]. One of the features of ACC is the expression of pancreatic enzymes, such as pancreatin, chymotrypsin, and lipase, which are stored in the larger cytoplasmic proenzyme granules [3]. Most patients have no specific symptoms, except those with excessive lipase secretion (about 15% of patients), who may develop symptoms of lipomemeitis such as joint pain, subcutaneous fat necrosis/nodules [3–5]. Thus, 50–60% of patients have advanced or metastasis disease at the time of diagnosis, resulting in a low survival rate and poor overall survival (OS) [4]. Another reason for the poor prognosis is the lack of standardized treatment for ACC. Despite the possibility of surgery as the only potential cure, ACC is often diagnosed late having already invaded blood vessels and surrounding tissue, and thus, radical resection is often impossible. Moreover, most patients relapse even with radical surgery [6]. The role of adjuvant chemotherapy and/or radiation therapy for patients with pancreatic ACC is yet unclear, and the effect of chemotherapy or radiotherapy alone is also elusive.

Due to the rarity of ACC, prospective studies are generally not readily available. Most of the existing studies are small samples, single-center retrospective case reports or case series [2, 4, 7–10], making it difficult to obtain meaningful hypotheses about the clinical characteristics and treatment of ACC patients. To the best of our knowledge, only three studies have reported ACC based on large public databases [11–13]. Wisnoski et al. [11] identified 672 patients with ACC from the Surveillance, Epidemiology, and End Results (SEER) database between 1973 and 2003. But since this study did not exclude patients diagnosed clinically, it might have overestimated the incidence of ACC. Besides, the variations of clinical characteristics overtime were not reported. Another recent study of the SEER database excluded most of the unknown data and the primary endpoint was cancer special survival [12]. All these three studies did not identify age-adjusted incidence (AAI) and age-adjusted survival (AAS). A recent study showed that married patients could get more spiritual and financial support from their partners, and thus had a longer survival period [14]. Even though the study has gradually attracted great attention, it lacked the information of marital status and failed to evaluate its role in patients with pancreatic ACC.

The present study used the recent data to (1) determine AAI and AAS for the first time; (2) update clinical features of ACC; (3) value efficiency of radiotherapy or chemotherapy in each stage; (4) determine the effect of marital status and other potential factors on OS.

## 2. Materials and methods

### 2.1 Study population

Data were obtained from the SEER database, which collects tumor clinicopathological information from population-based cancer registries covering nearly 34.6 percent of the U.S. population [15]. The SEER 18 Registries Research Data + Hurricane Katrina Impacted Louisiana Cases, November 2018 Submission (2000–2016) was used to calculate the incidence of ACC. The November 2018 Submission (1973–2016 varying) of SEER 18 Registries Custom Data (with additional treatment fields) was used to estimate the clinical feature. The two queues were created by SEER*Stat 8.36 software (Surveillance Research Program, National Cancer Institute SEER*Stat software version 8.3.6, www.seer.cancer.gov/seerstat).

The International Classification of Disease for Oncology, 3rd edition (ICD-O-3) was used to identify patients with pancreatic ACC (ICD-O-3 codes: 8550/3). Pancreatic ACC patients who were diagnosed between 2004 and 2016 by microscopic confirmation were selected. Besides, we included patients whose sequence numbers were marked as "one primary only", and those with active follow-up time. Finally, 252 patients of pancreatic ACC who met the inclusion criteria were included in the cohort.

### 2.2 Variables and endpoint

The following variables: age at diagnosis (≤66 years, >66 years), gender (female, male), race [black, white and others(American Indian/AK Native, Asian/Pacific Islander and Unknown)], marital status (married or unmarried, unknown), tumor size(≤3 cm, >3 cm, unknown), primary tumor location [head, body, tail, others(other specified parts of pancreas, overlapping lesion of pancreas, pancreatic duct), unknown], histology grade (well /moderately differentiated, poorly/undifferentiated, unknown), summary stage (localized, regional, distant, unstaged), surgery of primary tumors (yes, no, unknown), chemotherapy (yes, no/unknown), and radiotherapy (yes, no/unknown) were used to analyze the OS. Since the AJCC TNM staging system in the SEER database was only available between 2004 and 2015, we used the summary stage staging system available after 1998 in this research. The summary stages were divided into localized, regional, and distant disease. The localized disease was a tumor confined to the pancreas. Regional disease required the evidence of tumor invading adjacent structures that included duodenum, bile duct, ampulla of Vater, superior mesenteric vessels, hepatic artery, or locoregional lymph nodes. The distant disease referred to distant metastasis to other organs or lymph nodes beyond the local area. The primary endpoint of this study was OS, which is defined as the time from diagnosis to death.

### 2.3 Statistics analysis

The AAI and AAS (observed, expected, and relative survival rates.) was calculated using the SEER*Stat Software version 8.3.6. Survival probabilities were calculated by Kaplan-Meier and cum expected method using Ederer II. Continuous data were compared using student t-test while categorical data were compared by the chi-square test. Survival curves were drawn using the Kaplan–Meier method and differences among them compared by the log-rank test. Cox proportional hazards models were used to evaluate hazard ratios (HRs), 95% confidence intervals (CIs), and the factors that had independent predictive effects on the OS. Only factors that significantly affected the OS in the univariate Cox analysis were included in the multivariate Cox analysis. Statistical analysis was performed with MedCalc Statistical Software version 19.0.7 (MedCalc Software bvba, Ostend, Belgium; https://www.medcalc.org; 2019), and p < 0.05 was considered statistically significant.

## 3. Results

### 3.1 Incidence

**Fig 1:**
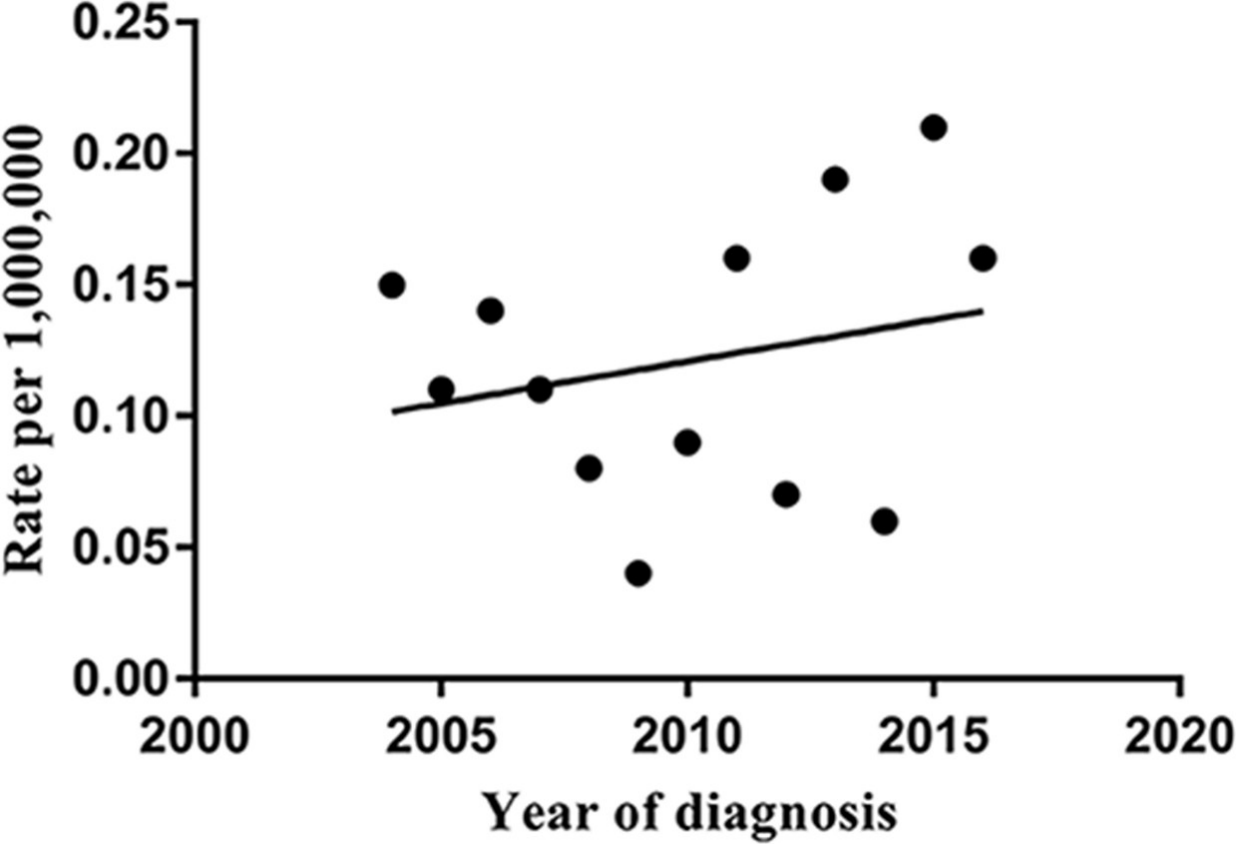
The age-adjusted incidence of pancreatic acinar cell carcinoma during 2004–2016.

The AAI of pancreatic ACC increased from 0.15 per 1000,000 in 2004 to 0.21 per 1000,000 in 2016, with a rising trend (Fig 1).

### 3.2 Clinical characteristics

A total of 252 eligible pancreatic ACC patients were identified in the study. The clinical features of the patients are shown in Table 1. The mean age at diagnosis was 63.79±14.79 years. The majority of the population was male (70.6%). The most common tumor sizes were >3cm (63.9%). Poorer differentiated (poorly/undifferentiated) tumors were observed in most patients (15.9%). Regarding the treatment, 75 (29.8%), 36 (14.3%), and 159 (63.1%) patients underwent primary surgery, radiotherapy, and chemotherapy, respectively.

**Table 1:** The clinicopathologic features of the patients with pancreatic acinar cell carcinoma.

Variable	Levels	Number (%)
**Age at diagnosis(years)**	Mean ± SD	63.79±14.79
	Median (range)	66(4–94)
	≤66	129(51.2)
	>66	123(48.8)
**Gender**	Male	178(70.6)
	Female	74(29.4)
**Race**	White	204(81.0)
	Black	25(9.9)
	Others	23(9.1)
**Material status**	Married	167(66.3)
	Unmarried	76(30.2)
	Unknown	9(3.6)
**Tumor size(cm)**	≤3	50(19.8)
	>3	161(63.9)
	Unknown	41(16.3)
**Location**	Head	108(42.9)
	Body	25(9.9)
	Tail	50(19.8)
	Others	26(10.3)
	NOS	43(17.1)
**Histological grade**	Well/ Moderately	31(12.3)
	Poorly/Undifferentiated	40(15.9)
	Unknown	181(71.8)
**Summary stage**	Localized	32(12.7)
	Regional	72(28.6)
	Distant	137(54.4)
	Unstaged	11(4.4)
**Lymph node metastases**	Negative	43(17.1)
	Positive	45(17.9)
	Unknown	164(65.1)
**Surgery**	Yes	75(29.8)
	No	175(69.4)
	Unknown	2(0.8)
**Radiotherapy**	Yes	36(14.3)
	No/Unknown	216(85.7)
**Chemotherapy**	Yes	159(63.1)
	No/Unknown	93(36.9)

The clinical features of the patients based on the stratification by the summary stage are shown in Table 2. The localized, regional and distant stage was 32, 72, 137, respectively. No significant difference was observed among patients in terms of age at diagnosis, race, marital status, and chemotherapy. However, compared with patients with the distant-stage disease, patients with localized and regional disease tended to underwent surgery, receive radiotherapy, have well /moderately differentiated tumors, have ≤3cm tumors and negative lymph node metastases (p<0.05). Furthermore, most patients with the localized disease were women. Patients with the regional disease were more likely to develop tumors in the head of the pancreas.

**Table 2:** The clinical features of the patients with pancreatic acinar cell carcinoma according to stratification by the summary stage.

Variable	Levels	Summary Stage			
		Localized(n = 32)	Regional(n = 72)	Distant(n = 137)	P-value
**Age at diagnosis(years)**	≤66	15(46.9)	37(51.4)	73(53.3)	0.804
	>66	17(53.1)	35(48.6)	64(46.7)	
**Gender**	Male	**16(50.0)**	55(76.4)	97(70.8)	0.024
	Female	**16(50.0)**	17(23.6)	40(29.2)	
**Race**	White	22(68.8)	59(81.9)	115(83.9)	0.234
	Black	4(12.5)	9(12.5)	12(8.8)	
	Others	6(18.8)	4(5.6)	10(7.3)	
**Marital status**	Married	19(59.4)	45(62.5)	96(70.1)	0.235
	Unmarried	13(40.6)	24(33.3)	35(25.5)	
	Unknown	0	3(4.2)	6(4.4)	
**Location**	Head	12(37.5)	**41(56.9)**	50(36.5)	0.088
	Body	3(9.4)	**6(8.3)**	15(10.9)	
	Tail	10(31.3)	**9(12.5)**	30(21.9)	
	Other	4(12.5)	**8(11.1)**	13(9.5)	
	NOS	3(9.4)	**8(11.1)**	29(21.2)	
**Histological grade**	Well/ Moderately	**8(25.0)**	**12(16.7)**	11(8.0)	<0.001
	Poorly/Undifferentiated	**2(6.3)**	**18(25.0)**	17(12.4)	
	Unknown	**22(68.8)**	**42(58.3)**	109(79.6)	
**Tumor size (cm)**	≤3	**9(28.1)**	**19(26.4)**	20(14.6)	<0.001
	>3	**21(65.6)**	**52(72.2)**	85(62.0)	
	Unknown	**2(6.3)**	**1(1.4)**	32(23.4)	
**Lymph node metastases**	Negative	**18(56.3)**	**19(26.4)**	6(4.4)	<0.001
	Positive	**0**	**26(36.1)**	19(13.9)	
	Unknown	**14(43.8)**	**27(37.5)**	112(81.8)	
**Surgery**	Yes	**21(65.6)**	**41(56.9)**	13(9.5)	<0.001
	No/Unknown	**11(34.4)**	**31(43.1)**	124(90.5)	
**Radiotherapy**	Yes	**6(18.8)**	**21(29.2)**	7(5.1)	<0.001
	No/Unknown	**26(81.3)**	**51(70.8)**	130(94.9)	
**Chemotherapy**	Yes	17(53.1)	51(70.8)	89(65.0)	0.216
	No/Unknown	15(46.9)	21(29.2)	48(35.0)	

Statistically significant differences between other summary stage disease and distant disease results are shown in bold (P < 0.05).

### 3.3 Survival analysis

The survival rates of ACC diagnosed between 2004 to 2016 are shown in Table 3. The observed survival refers to the survival rate caused by all causes of death. The relative survival rate refers to the survival rate caused by the tumor while the expected survival rate refers to the death rate caused by the tumor. The observed survival rate of 1-year, 2-year, and 5-year were 53.5%, 34.6%, 17.5%, respectively.

**Table 3:** Observed, expected and relative survival rates of pancreatic acinar cell carcinoma patients from 2004 to 2016.

Years	Observed survival (SE)	Expected survival	Relative survival (SE)
1	53.5% (3.3%)	97.5%	54.9% (3.3%)
2	34.6% (3.3%)	95.2%	36.4% (3.1%)
3	26.9% (3.2%)	93.3%	28.8% (3.0%)
4	20.3% (3.0%)	91.4%	22.1% (2.8%)
5	17.5% (2.9%)	89.1%	19.6% (2.7%)

**Fig 2:**
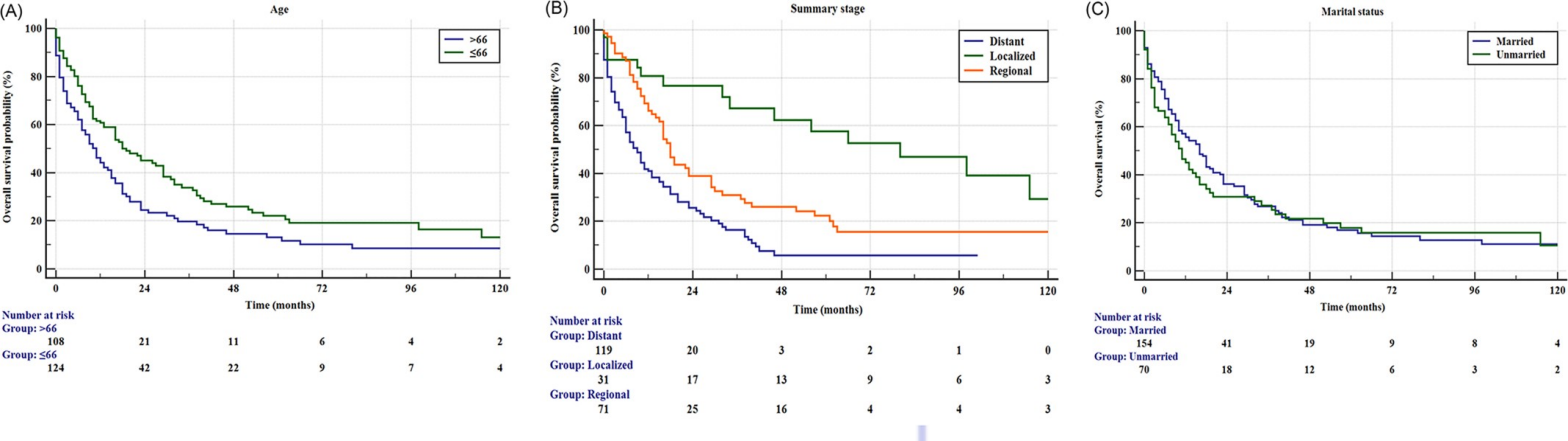
(A) Kaplan–Meier estimated overall survival (OS) for patients with pancreatic acinar cell carcinoma (ACC) in terms of age (p = 0.003), (B) Kaplan–Meier estimated OS for patients with pancreatic ACC in terms of summary stage (p<0.001 for both); (C). Kaplan–Meier estimated OS for patients with pancreatic ACC in terms of marital status (p = 0.448).

The Kaplan–Meier estimated survival curves in terms of age are shown in Fig 2A. The older patients had a poorer prognosis than younger patients (p< 0.001). As shown in Fig 2B, patients with pancreatic ACC presenting with localized stage had better OS than patients with regional stage and distant stage (all p<0.001). The effect of marital status on OS is shown in Fig 2C. We found that the risk of death in unmarried patients with pancreatic ACC was roughly the same as in married patients, with no statistically significant difference.

**Fig 3:**
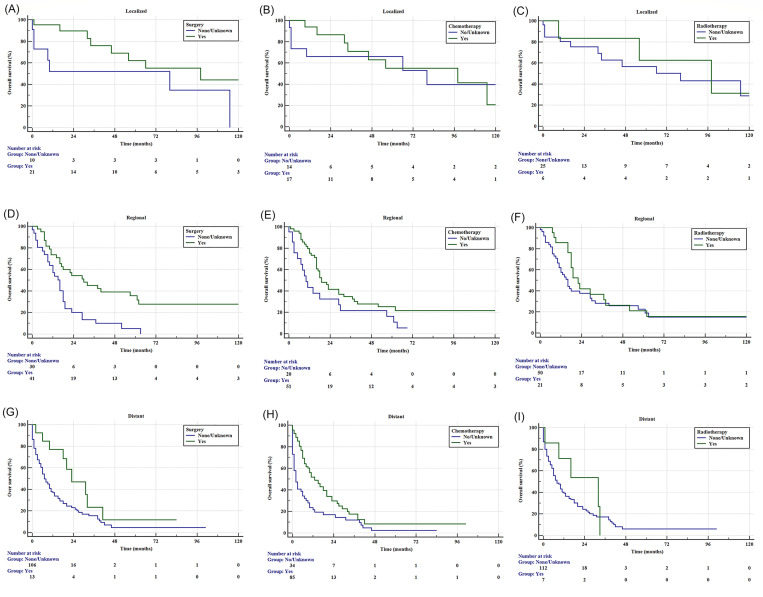
Kaplan–Meier estimated overall survival for patients with pancreatic acinar cell carcinoma based on the summary stage. (A-C): based on localized stage, A:surgery (p = 0.053), B: chemotherapy (p = 0.609), and C: radiotherapy (p = 0.729), (D-F): based on regional stage, D:surgery (p<0.001), E: chemotherapy (p = 0.041), F: radiotherapy (p = 0.369) (G-I) based on distant stage:G: surgery (p = 0.031), H: chemotherapy (p<0.001), I: radiotherapy (p = 0.365).

The Kaplan-Meier survival curve stratified according to the treatment and summary stage is shown in Fig 3. It was observed that patients who underwent primary surgery had significantly better OS than those who did not in the regional and distant stage, but not in the localized stage. Chemotherapy was revealed as an effective treatment for the regional and distant disease, but not the localized disease. Radiotherapy showed no survival benefit in all stages.

Factors potentially influencing OS were further analyzed using Cox proportional hazards analysis and are shown in Table 4. In univariate analysis, older age, positive lymph node, absence of treatment were associated with poor prognosis (HR>1, p<0.05), while the female gender and locoregional stage were the favorable variables (HR<1, p<0.05). Notably, marital status was not an independent predictor for prognosis. In order to adjust the confounding factors, variables with significant influence on OS in univariate Cox regression were included in the multivariate analysis. We found that patients who present with older age and later summary stage were independent adverse prognosis factors for ACC. Similarly, failure to receive surgery and chemotherapy were also independent adverse prognosis factors. Compared with those who received surgery, the risk of death without surgery increased by 1.784 times (HR = 2.784, 95%CI: 1.397–5.551). On the other hand, the risks in patients without chemotherapy increased to 1.865 times (HR = 1.865, 95%CI: 1.341–2.592).

**Table 4:** Univariate and multivariate regression analysis of overall survival in patients with pancreatic acinar cell carcinoma.

Variable	Levels	Univariate		Multivariate	
		HR (95%CI)	P-value	HR (95%CI)	P-value
**Age at diagnosis(years)**	≤66	1		1	
	>66	1.533(1.146–2.050)	0.004	1.569(1.151–2.138)	0.004
**Gender**	Male	1		1	
	Female	0.683(0.487–0.957)	0.027	0.849(0.597–1.208)	0.363
**Race**	White	1		/	/
	Black	1.143(0.723–1.806)	0.568	/	/
	Others	0.640(0.363–1.129)	0.124	/	/
**Material status**	Married	1		/	/
	Unmarried	0.864(0.380–1.962)	0.726	/	/
	Unknown	1.122(0.819–1.537)	0.472	/	/
**Tumor size(cm)**	≤3	1		/	/
	>3	1.188(0.803–1.757)	0.389	/	/
	Unknown	2.371(1.464–3.842)	<0.001	/	/
**Location**	Head	1		/	/
	Body	0.845(0.487–1.467)	0.549	/	/
	Tail	0.710(0.467–1.080)	0.110	/	/
	Other	1.169(0.722–1.891)	0.525	/	/
	NOS	1.628(1.101–2.407)	0.015	/	/
**Histological grade**	Well/ Moderately	1		/	/
	Poorly/Undifferentiated	1.354(0.786–2.334)	0.275	/	/
	Unknown	1.436(0.920–2.240)	0.111	/	/
**Lymph node**	Negative	1		1	
**metastases**	Positive	2.200(1.278–3.785)	0.004	1.492(0.794–2.804)	0.214
	Unknown	3.403(2.145–5.398)	<0.001	1.064(0.482–2.350)	0.879
**Summary stage**	Distant	1		1	
	Localized	0.243(0.139–0.427)	<0.001	0.338(0.185–0.620)	<0.001
	Regional	0.543(0.388–0.760)	<0.001	0.786(0.530–1.167)	0.232
	Unknown	1.396(0.729–2.675)	0.315	0.906(0.456–1.799)	0.777
**Surgery**	Yes	1		1	
	No/Unknown	3.181(2.226–4.546)	<0.001	2.784(1.397–5.551)	0.004
**Radiotherapy**	Yes	1		1	
	No/Unknown	1.653(1.089–2.508)	0.018	1.221(0.762–1.956)	0.406
**Chemotherapy**	Yes	1		1	
	No/Unknown	1.787(1.332–2.398)	<0.001	1.865(1.341–2.592)	<0.001

## 4. Discussion

Pancreatic ACC is a rare clinical disease understudied. The currently published literature includes several case reports and a relatively small number of clinical case series. In this study, we used the SEER database, a large sample of data from multiple centers that is well represented, to study the incidence, clinical characteristics, and prognostic factors of ACC.

### 4.1 Contemporary worsening survival trends

Due to the rarity of this tissue type, few studies have reported AAI. To our knowledge, this is the first population-based study to report the incidence of primary pancreatic ACC. Based on our results, the AAI of pancreatic ACC increased from 0.15 per 1000,000 in 2004 to 0.21 per 1000,000 in 2016, with an elevatory trend. The increased incidence could be due to the improved diagnostic techniques that have allowed for differentiation of some ACCs that were previously misdiagnosed as adenocarcinoma or neuroendocrine tumor [16].

Although previous studies have shown that ACC has a better prognosis than adenocarcinoma, we found that the prognosis of ACC was deteriorating. In our study, the 1-year, 2-years, and 5-years survival rates for pancreatic ACC patients were 53.5%, 34.6%, and 17.5%, respectively, which were significantly lower than the previously reported rates (78.5%, 67.0%, and 42.8%) [17]. We also found more patients presenting with a distant stage than in a previous study (54.4% vs 53.1%). The fact that the previous study had more patients with the unknown stage of ACC than the present study (8.8% vs 4.4%), which could make existing differences even more pronounced. From the above evidence, a deterioration in the survival trend could be seen. The current trend of ACC progression is noteworthy and require more frequent follow-up and aggressive and timely treatment. However, since our study is retrospective, this conclusion might need to be interpreted with caution. For instance, our study had more patients in the distant stage than the research conducted by Wisnoski et al. [17], but the difference was not significant and might be due to other errors. However, the decline in survival rates was significant and certainly could not be due to error.

Our study found that patients diagnosed with localized or regional disease had a more significant survival benefit and better clinical characteristics than patients with distant disease. Similar to previous research, we found the distant-stage to be an independent risk factor. The risk of death in the local stage was significantly reduced by 0.662 times compared with distant-stage. Although the risk of death in the regional stage was also reduced by 0.214 times compared with the distant stage, it was not statistically significant. In other words, the diagnosis in the early stage showed a good prognosis signifying its importance. Since the most patients diagnosed with localized diseases in this study were found to be of the female gender, it is more likely for women to be diagnosed with localized disease.

### 4.2 Older patients were associated with poorer prognosis

In our study, the median age of patients with pancreatic ACC between 2004 and 2016 was 63.79±14.79 years, which was younger than the median age of 70.2±12.5 years reported by Wisnoski et al. from 1988 to 2003 [17], and similar to that reported by He et al. [12]. The phenomenon suggesting that the population of patients with ACC is getting younger to some extent. This may be associated with improved diagnostic techniques, as well as increased awareness of physical examination, leading to easier detection of ACC.

Our results of the survival curve showed that elderly patients were generally associated with poorer prognosis compared to younger patients (p<0.01), consistent with previous studies [12]. Multivariate analysis showed 1.569 folds in mortality in older patients compared to younger patients. The reason for the poor prognosis of the elderly patients may be that with the increase of the patient's age, the immune system declines, and the patient's physical function declines [18], which is not conducive to the treatment of tumor. Our study also shows that patients who undergo surgery are younger (S1 Table).

### 4.3 Married patients had a better prognosis

The effect of marital status on tumor prognosis is increasingly being recognized [19–21], and it has been shown that unmarried patients have a much higher risk of metastatic cancer, undertreatment, and cancer death than the married patients [14]. This could be because the unmarried patients do not receive the same psychological and financial support from their partners as the married patients do [22]. Thus, they took a more negative approach to deal with their own tumors and have a worse physical state. Similarly, marital status is considered as one of the important prognostic factors for pancreatic ductal adenocarcinoma and pancreatic neuroendocrine neoplasms [23, 24]. However, the past studies on the effect of marital status on pancreatic masses did not include ACC.

In this study, we explored for the first time (to the best of our knowledge) the impact of marital status on the prognosis of ACC. Compared with unmarried patients, we found no significant increase in the OS of married patients (p> 0.05). However, this finding was contrary to the previous conclusion. For confirmation of this result, we compared baseline characteristics of married and unmarried groups (S2 Table) and found a difference between gender and chemotherapy in married patients and unmarried patients. Most married patients were men and received chemotherapy. Past studies have shown that chemotherapy can benefit patients [6, 25, 26], and men might be more tolerant of higher intensity than women. Therefore, we believe that married patients have a better prognosis than unmarried patients, and our contradictory conclusions could have been caused by a small sample size.

Most patients with ACC in our study had a poor histological grade. Although tumor grade is considered an important prognostic factor for adenocarcinoma [27], we did not find a significant effect of histological grade on OS in patients with ACC (p>0.05). Similar to other studies [12, 13, 28], tumor size had no significant effect on OS even though ACC unusually presents with larger tumors.

### 4.4 More radical management methods may achieve longer survival

No randomized controlled trials have been reported for the treatment of ACC, as most of the studies are based on case reports and case series. Currently, the treatment methods of adenocarcinoma including surgery, radiotherapy, and chemotherapy are usually applied for the clinical treatment of ACC. If no signs of metastasis are found in the preoperative evaluation, surgical treatment, the only potential cure, is usually recommended. In this study, the 5-year survival rate for patients undergoing surgery was 11.7%, compared to 4.7% for patients without surgery (p<0.001). Similarly, the 5-year survival rate was significantly improved in patients who present with local-regional disease receiving surgery [17, 29]. Several studies also found a significant increase in OS among patients with IV stage receiving surgical resection [29, 30]. This suggests that when evaluating whether ACC could be removed, the criteria should be relaxed to allow for more aggressive surgery to achieve tumor resection. Although there was a significant improvement in OS in patients undergoing surgery at any stage, the postoperative recurrence rate is as high as 42–72% [8]. Furthermore, the most common pattern of recurrence in ACC is distant metastasis [28], hence the necessity to adopt more active adjuvant therapy strategies after the operation.

Unlike previous studies based on the public database, we included the information on chemotherapy and evaluated its effects on ACC. Chemotherapy was the most commonly used treatment, with 63.1% of patients receiving it. The survival curve showed that patients who received chemotherapy had a longer survival in regional and distant stages. And multivariate cox regression analysis also showed that receiving chemotherapy was a favorable factor for prognosis. These results were consistent with other studies [6, 25, 26]. However, no consensus has been reached on the current first-line chemotherapeutic regimen for patients with ACC. It might be necessary to test for the BACR2 gene as patients with a mutation in the BACR2 gene could benefit from platinum-based drugs and even survive for more than five years [16, 31], Furthermore, 53.1% of patients received adjuvant chemotherapy in the local stage proving that ACC might need more aggressive treatment from another aspect.

Only 36 patients (14.3%) received radiotherapy with respective 6, 21, 7 patients in localized, regional, distant disease. Additionally, the survival curve revealed that radiotherapy did not have a survival benefit in each stage. Considering surgical treatment, most of the patients undergoing surgery are young, and only a few of them receive secondary radiotherapy. This implies that the proportion of elderly patients receiving radiotherapy could be higher, which might lead to no survival difference after radiotherapy. Also, the low statistics caused by the limitation of data quantity could explain this result. We, therefore, applied the cox risk models to correct the confounding factors, we did not find that radiotherapy improved the risk of death. In fact, radiotherapy is rarely applied alone to a tumor in clinical practice and is usually used in combination with chemotherapy or surgery. Survival benefits are usually achieved by combining radiotherapy with chemotherapy or surgery in patients with unresectable advanced disease [32–34].

In summary, ACC appears to be more inert than adenocarcinoma, thus require different management measures. More extensive surgical management, such as surgical resection or chemoradiotherapy at the stage of distant metastasis, and more aggressive medical treatment are also needed to avoid postoperative recurrence.

### 4.5 Limitation

Our research had some limitations. Firstly, the SEER database lacks some detailed data such as surgical margin, radiation dose, chemotherapy, and regimen which somehow limited the analysis. Secondly, ACC often incorporates other endocrine components [35]. Although we excluded patients with acinar cystadenoma, there is no centralized pathologist in the SEER database, hence a possibility of cancer misclassification. Thirdly, we used the summary stage, which is not intuitive, because the TNM staging system currently included in the SEER database is only suitable for 2004–2015. Finally, although this study investigated a large number of patients with pancreatic ACC, the overall number was still limited, and some clinical data were missing, which could also affect our conclusion.

## 5. Conclusions

Pancreatic ACC is a highly malignant tumor with an increasing incidence in recent years. The rate of distant metastasis is increasing, and the survival rate is worsening with time. Therefore, there is a need for more aggressive treatment and follow-up. From our results, married patients have a better prognosis, while elderly patients, those who have not received surgery, and those who have not received chemotherapy have a poor prognosis. Both surgery and chemotherapy are effective means of treatment. Radiotherapy combined with surgery or chemotherapy is also one of effective means. Further studies are needed to verify the effects of surgery, radiotherapy, and chemotherapy on ACC.

## Supporting information

S1 Table The clinical features of the patients with pancreatic acinar cell carcinoma according to stratification by the surgery or not. (XLSX)

S2 Table The clinical features of the patients with pancreatic acinar cell carcinoma according to stratification by the marital status. (XLSX)

S1 Data Raw data on the clinical characteristics of all patients with acinar cell carcinoma. (XLSX)

S1 File The certificate of English editing. (PDF)
