# Supplementary material for: The contemporary trend in worsening prognosis of pancreatic acinar cell carcinoma: A population-based study
Source: PLoS One. 2020 Dec 17;15(12):e0243164. doi: 10.1371/journal.pone.0243164 (PMC7746196; doi:10.1371/journal.pone.0243164)
Supplement: S1 File — (PDF) [file pone.0243164.s004.pdf]

Freescience Editorial Team

Certificate of English Editing

Paper Title

The contemporary trend in worsening prognosis of pancreatic acinar cell carcinoma: A population-based study

Authors

Duorui Nie, Bin Shi, Tao Zhang,Chuyao Chen, Chongkai Fang, Zhijun Yue, Peng Wu, Zhiming Wu, Xuewu Huang, Meng Li

This certificate is issued as a confirmation that the paper mentioned above has been proofread and edited for language clarity and grammar by professional editors of our company.

We guarantee that the original message was not distorted, and that the paper is understandable and free of errors assuming that the changes and suggestions given are accepted, and text is not altered without our knowledge

Date of Editing: 08-27-2020
